# Protein Prenylation Makeovers in Plants: Insights into Substrate Diversification

**DOI:** 10.3390/ijms262110638

**Published:** 2025-10-31

**Authors:** Quentin Chevalier, Pauline Debié, Alexandre Huchelmann, Andréa Hemmerlin

**Affiliations:** Institut de Biologie Moléculaire des Plantes, CNRS, Université de Strasbourg, 12 rue du Général Zimmer, F-67084 Strasbourg, Francepauline.debie2@etu.unistra.fr (P.D.);

**Keywords:** *Arabidopsis*, biosynthesis pathway, CaaX motif, protein prenylation, farnesyl diphosphate, geranylgeranyl diphosphate, methylerythritol phosphate, mevalonate, prenyltransferases, regulation

## Abstract

Type-I protein prenylation, the post-translational modification of CaaX motif-containing proteins, relies on two substrates: the target protein and a mevalonate-derived prenyl diphosphate co-substrate, either farnesyl diphosphate (FPP) or geranylgeranyl diphosphate (GGPP). Two enzymes, protein farnesyltransferase and type-I geranylgeranyltransferase, recognize and bind both co-substrates. Modifying potentially hundreds of distinct protein targets within a constrained timeframe poses a major regulatory challenge for the cell. However, the mechanisms controlling prenyltransferase activity, including substrate availability, enzyme specificity, and catalytic efficiency, remain poorly understood, particularly in plants. Plant prenylation systems exhibit distinctive features. The diversity of prenyl diphosphate donors is expanded by the plastidial methylerythritol phosphate pathway, which supplements the mevalonate pathway and may provide alternative prenyl groups beyond the canonical FPP and GGPP. Additionally, many CaaX-containing proteins are plant-specific, and post-transcriptional modifications generate multiple prenylatable isoforms, increasing substrate complexity. In this review, we examine the diversification of both prenyl diphosphate donors and protein substrates in plants, hypothesizing that such diversification may illuminate key mechanisms underlying the cellular regulation of protein prenylation.

## 1. Modifications of Proteins by Prenyl Groups

### 1.1. Generalities

Prenyl group modification represents a ubiquitous biochemical process found across all branches of life. These so-called prenylations, or isoprenylations, are enzyme-catalyzed reactions calling for different classes of prenyltransferases (PTs) [1]. Accordingly, PTs promote the formation of new C-C or C-S bonds and recognize all along two different substrates: a prenyl diphosphate and the acceptor molecule being either another prenyl group (e.g., geranyl diphosphate), nucleic acids (e.g., tRNA), specialized metabolites (e.g., flavonoids), or amino acids (e.g., RAS (RAt Sarcoma) small guanosine 5′-triphosphatases (GTPase)). This review focusses on type I prenylation of CaaX-motif-containing proteins, a post-translational modification restricted to eukaryotic cells, but several viral and bacterial pathogens encode protein substrates that can undergo prenylation using the host enzyme machinery [2,3]. Rather than reiterating existing comprehensive overviews of plant protein prenylation [4,5,6,7,8], this review provides a biochemical perspective on the potential in vivo regulatory mechanisms of this process through the analysis of substrate diversification. To set the stage, we begin with a brief overview of the protein prenylation process to provide the necessary context for this discussion.

**Figure 1:**
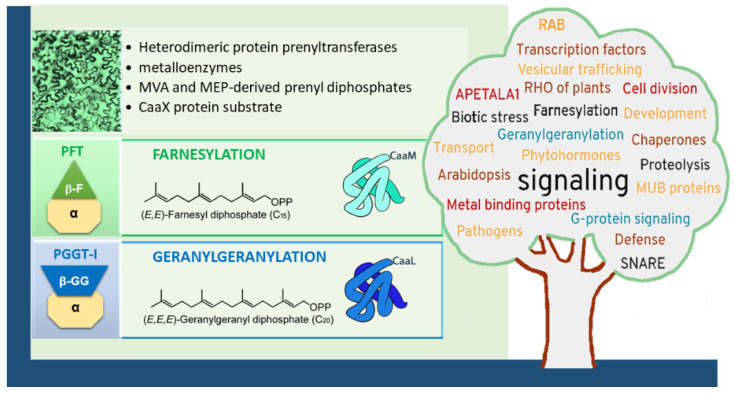
Type-I protein prenylation in plants (here represented by epidermic cells in a tobacco leaf, visualized in the top left corner) and biological functions. Heterodimeric protein prenyltransferases, composed of a common α-subunit and a specific β-subunit, catalyze the lipid modification of target proteins in a sequential manner. First, the enzyme complex binds a prenyl diphosphate donor (either FPP or GGPP), followed by the recognition of a protein substrate containing a CaaX motif. This results in the formation of a cysteinyl–prenyl thioether bond between the cysteine residue of the CaaX motif and the prenyl group. The identity of the terminal residue in the motif determines the type of prenylation: CaaM motifs typically lead to farnesylation, whereas CaaL motifs favor geranylgeranylation. Following prenylation, some substrates undergo two additional post-isoprenylation modifications to become fully functional. These include: (1) endoproteolytic cleavage of the terminal “aaX” tripeptide, and (2) reversible carboxymethylation of the now-terminal prenylated cysteine residue [5]. These subsequent steps are not depicted in the Figure. Protein prenylation is required for proteins involved in many biological functions [5,6,7,8], as indicated on the word cloud shaped like a tree and constructed with https://www.nuagesdemots.fr/, accessed on 21 February 2021) by using key-words collected in the literature addressing topics related to plant protein prenylation.

CaaX motif (C = cyst; a = aliphatic; X = C-terminus amino acid) proteins are modified by prenyl moieties via thioether linkages to cysteines located in the fourth position of the carboxyl terminus [9]. In general, modified proteins acquire new functionalities, such as a gain in hydrophobicity. This, in turn, promotes interactions with membrane structures or other proteins. Additionally, prenylation can induce conformational rearrangements in the three-dimensional structure, as demonstrated for the peroxisomal import receptor PEX19 [10]. Undoubtedly, this lipid modification plays a fundamental role in numerous biological processes, such as various signaling pathways, cellular trafficking, cell proliferation, and apoptosis. To list just a few examples described in plants [4], prenylated proteins function in phytohormone signaling or inducible specialized metabolite production, in defense mechanisms against pathogens or abiotic stresses, in vesicular transport, cellular polarity or meristem development (Figure 1) [5,6,7,8]. In addition to these functions, the agronomic potential of plants deficient in protein PTs (PPTs) activity has also been highlighted, notably through enhanced tolerance to drought stress [11,12]. Once modified, a number of proteins undergo further post-prenylation maturation events, including aaX peptide proteolysis and cysteinyl carboxymethylation [13]. Methylation neutralizes the negatively charged, hydrophilic cysteinyl residue to an uncharged, hydrophobic group, thereby increasing membrane affinity by an additional 2 to 10-fold [14]. Together, the whole procedure enables significant affinity to membranes, positioning prenylation as the leading lipid modification of proteins in terms of Kd affinity constants [15]. Thus, post-maturation steps have also biological functions and the methylation step is the only reversible step in the process, with isoprenylcysteine α-carbonyl methylesterase (ICME, EC 3.1.1.n2) catalyzing the removal of the methyl group [16].

Although protein lipidation is necessary for membrane anchoring, it is not sufficient on its own to ensure specific membrane targeting. Additional factors, such as the presence of a positively charged polybasic domain, like that found in Rho Of Plants (ROP) proteins, play a crucial role. These domains interact with anionic phospholipids, such as phosphatidylinositol 4-phosphate (PI4P), phosphatidic acid (PA), and phosphatidylserine (PS), generating strong electrostatic fields at the plasma membrane [17]. Moreover, membrane composition can further influence protein binding; for instance, the incorporation of sterols into negatively charged membranes alters protein interactions [18]. Therefore, a comprehensive understanding of membrane biochemistry, including its composition and dynamics, is essential for elucidating the localization and function of prenylated proteins.

Uncovering the fine-tuned biochemical processes that regulate protein prenylation is essential for anticipating and potentially controlling the adaptive mechanisms organisms may activate in response to impaired prenylation. *Arabidopsis* plants remain viable even when genes encoding individual PPT subunits are deleted; a stark contrast to other organisms, in which loss-of-function mutations in PPT are typically lethal. However, it is important to emphasize that viability in *Arabidopsis* depends on maintaining optimal growth conditions, particularly with respect to photoperiod and light quality [19]. This conditional viability might explain findings from a recent publication on rice, which reported that not all knockout (KO) deletion mutants are viable under standard conditions [20]. The phenotypic plasticity reflects the evolution of unique compensatory mechanisms in plants to modify proteins with lipid moieties, opening the door to uncovering novel modes of regulating protein prenylation. It also suggests that the plant prenylome (the complete set of prenylated proteins) is more adaptable or functionally redundant, enabling compensation for the loss of specific prenylation activities.

### 1.2. Protein Prenyltransferases

**Figure 2:**
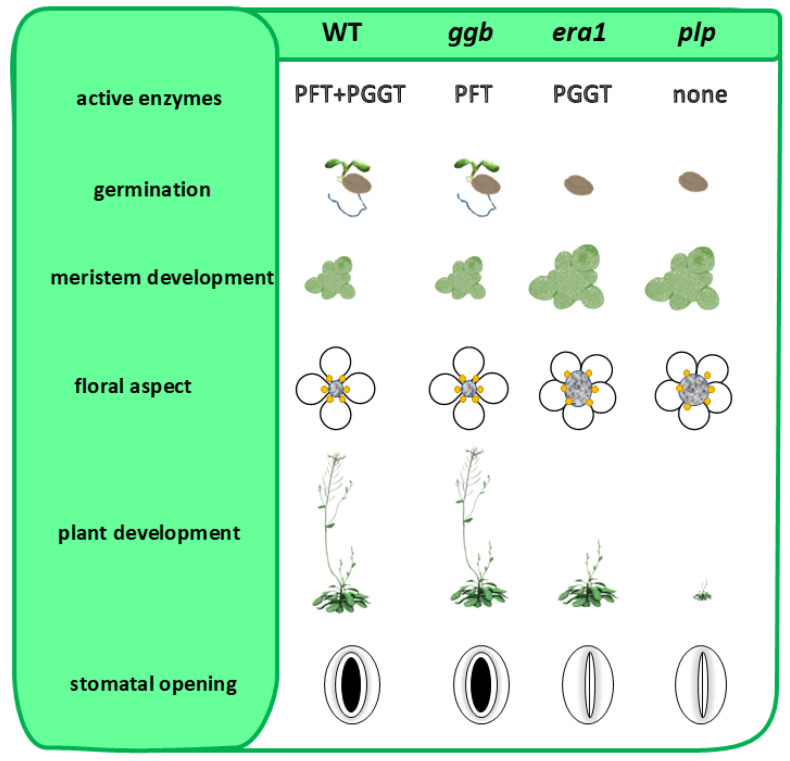
Phenotypic traits of loss-of-function mutant lines in genes encoding protein prenyltransferases subunits in *Arabidopsis thaliana*. Compared are wild type (WT), *ggb* (T-DNA insertional mutant in the β-GG subunit: locus *At2g39550*), *era1* (T-DNA insertional mutant in the β-F subunit: locus *At5g40280*) and *plp* (T-DNA insertional mutant in the α-subunit: locus *At3g59380*). Absence of PGGT-I catalyzed protein prenylation has only low impact on *Arabidopsis* phenotypic traits (germination and plant development delay; meristem size and floral aspect and stomatal opening). This suggests a high substrate plasticity of PFT enzyme activity.

Type-I PPTs consist of two heterodimeric metalloenzymes that are Zn^2+^- and Mg^2+^-dependent, namely protein-farnesyltransferase (PFT; EC 2.5.1.58) and type I protein-geranylgeranyltransferase (PGGT-I; EC 2.5.1.59). Both enzymes share a common α-subunit (locus *At3g59380* from *Arabidopsis thaliana*), but distinct β-subunits (β-F subunit: locus *At5g40280* and β-GG subunit: locus *At2g39550*) that provide substrate selectivity (Figure 1). As mentioned above, plants are unique in that deletion mutants of protein prenylation remain viable, whether involving the α-subunit (*plp* mutant in *Arabidopsis*) or β-subunits (*era1* and *ggb2* mutants) via T-DNA insertions [21,22,23,24]. However, these mutants display distinct phenotypes, with *era1* and *plp* mutant lines showing the most pronounced divergences from the wild type (WT) (Figure 2). Specifically, they exhibit delayed seed germination, enlarged floral meristems, flowers with increased petal numbers and abnormal organ development, as well as overall delays in plant growth and development. These observations suggest that protein farnesyltransferase (PFT), the only functional prenylation enzyme remaining in a *ggb2* background, is likely the most versatile in substrate recognition, and may explain why *ggb2* KO mutants maintain phenotypes closer to *Arabidopsis* WT compared to *era1* and *plp* mutant lines.

Other types of protein prenylation have been described in eukaryotes. A second class of enzymes (PGGT-II; EC 2.5.1.60) catalyzes the modification of RAB proteins on two cysteines using 2 geranylgeranyl groups. The enzyme imposes an RAB escort protein (REP) to be associated with the RAB substrate, which is more challenging for the study of kinetic properties of the enzyme. Fifty-seven loci that encode RAB GTPases have been identified in the *Arabidopsis thaliana* genome [25]. These Ras-like, small GTPases function in targeting and/or tethering transport vesicles to acceptor compartments [25]. Some proteins can serve as substrates for more than one type of PPT, highlighting the flexibility of the prenylation system. For example, in humans that RAB8 is modified by type I and type II PGGT [26]. Along the same lines, it has been shown that *Arabidopsis* RAB-PGGT-II is capable of modifying several CaaX-containing proteins in vitro [27]. A third class of enzymes has been described in animals. It is proposed that this family catalyzes the transfer of a geranylgeranyl group onto preliminarily farnesylated proteins such as SNARE complex protein YKT6 (UniProtKB-O15498) or FBXL2 (UniProtKB-Q9UKC9), both of which possess an additional cysteine, resulting in a CCaaX motif [28,29,30,31]. In plants, the CCaaX motif of YKT6 is conserved as seen in *Arabidopsis* locus *At5g58060*. However, it has recently been reported by using an acyl-RAC assay that AtYKT61 is *S*-acylated [32]. In *Plasmodium falciparum*, a parasite evolutionary closely related to algae, YKT6.2 is both prenylated and myristoylated, whereas YKT6.1 is only prenylated [33]. Other proteins, including RAB family members and AtYKT62, may also undergo similar modifications; however, direct experimental evidence remains lacking. In addition, Shi et al. [27] proposed that a protein prenyltransferase α-subunit-like (PPAL; *At1g10095*), although truncated, could nevertheless substitute for the canonical α-subunit in *Arabidopsis* under specific conditions [34]. More recently, Suazo and coworkers [35] characterized a modification through esterification of an aldehyde dehydrogenase ALDH9A1. They proposed that this reversible modification originated from endogenous farnesal or geranylgeranial [35], the latter obtained by the FPP salvage machinery [36]. Altogether, it is estimated that approximately 1–3% of the eukaryotic proteome undergoes prenylation, underscoring its broad functional relevance. A temporal, or even spatiotemporal dimension, likely governs the regulation of protein substrates involved in signaling processes, reflecting their dynamic behavior and the need for turnover. The coexistence of at least four distinct PPTs underscores the complexity of selectively modifying specific proteins with particular prenyl groups or unique combinations of lipid moieties in eukaryotic cells.

In contrast to plants, the enzymology of animal and fungal proteins is well-defined and extensively characterized, primarily through in vitro studies [37]. Beyond CaaX protein substrates, all PPTs commonly utilize allylic prenyl diphosphates (C_15_-farnesyl diphosphate, FPP or C_20_-geranylgeranyl diphosphate, GGPP) as co-substrates (Figure 1). In animal cells, FPP and GGPP are products of mevalonic acid (MVA) [38], while in plants, in addition, the plastidial methylerythritol phosphate (MEP) pathway contributes as well to their biosynthesis [39,40,41,42,43,44,45]. The resolution of non-plant PFT and PGGT-I three-dimensional structures reveals a clear substrate preference of FPP for PFT and GGPP for PGGT-I, and provides detailed molecular insight into the mechanism of prenyl group transfer (details reviewed by Jiang et al. [46]). Mammalian PFT exhibits ~30-fold higher affinity for FPP than for GGPP, while PGGT-I binds GGPP ~300 times more tightly than FPP [47]. Structural and mechanistic studies suggest that prenyl diphosphate precursors (FPP or GGPP) are primarily requisitioned by PPTs and bound into a funnel-like hydrophobic cavity, including the active site. In a second step, the CaaX protein substrate binds the prenyl diphosphate-PPT enzyme complex [48,49,50]. As mentioned, PPTs are metallo-enzymes with thiolate activation requiring Zn^2+^ coordination, and diphosphate stabilization requiring Mg^2+^ [49]. The release of the modified protein product requires the binding of a new prenyl diphosphate into the active site [51]. In plants, as in other organisms, the characterization of enzymes in vitro has revealed distinct catalytic characteristics [52]. Unlike PGGT-I, recombinant *Arabidopsis thaliana* PFT unveils little specificity for its C-terminus X amino acid in vitro, which allows cross-reactivity with PGGT-I function in vivo. These cross-reactivities are now well accepted and well documented, particularly for human and animal enzymes [53]. At the same time, under restrictive conditions in vivo, alternative prenylation occurs. For instance, during *Plasmodium falciparum* infections and in cancer cells, following therapeutic treatments, acquisitions of resistance to drugs targeting PFT have been described [54,55]. This proves that PGGT, or another enzyme, takes over, when needed, the modification of important proteins *in cellulo*. Furthermore, the ability of *Arabidopsis* *era1*, *ggb2* and *plp* knockout mutants to survive under standard growth conditions suggests that this is also true for plants. Taken together, these observations indicate a notable metabolic flexibility in protein prenylation.

In addition to flexible substrate recognition, PPT activity can also be modulated by post-translational modifications (PTMs), with, for example, phosphorylation of the α-subunit activating the enzyme in mouse fibroblasts and adipocytes following activation with insulin [56]. It has been proposed that phosphorylation of the α-subunit functions as a stabilizer of the β-subunit with a lower affinity when phosphorylated [57]. More recently, it has been demonstrated that phosphorylation modulates the catalytic site by rearranging interactions between FPP and the protein substrate catalytic Zn^2+^ ion/coordinating residues and hot-spot residues at the interface of the subunits, all of which led to the stabilization of the substrate and facilitation of the release of the product, thus collectively expediting the reaction rate [58]. Interestingly, when cell-free plant crude extracts are taken as enzyme source, apparent activity of PFT is found to be higher than that of PGGT [40,52,59]. But surprisingly, incorporation rates into protein fractions of radiolabeled farnesol (Fol) after feedings are much lower than those with geranylgeraniol (GGol) [60]. Such observations suggest either that the PGGT enzyme is unstable in vitro and activity is partially lost and underestimated, or that prenylation dynamics in vivo are different from those in vitro, with, for instance, both enzymes being capable of modifying a specific protein under specific circumstances. This is in support of the view that the substrate specificities assessed in vitro, using purified enzymes, do not necessarily reflect the regulation of enzyme activities in cellulo.

## 2. Prenyl Diphosphate Substrates

### 2.1. A Double Metabolic Origin in Plants

Plants must carefully allocate metabolic resources to maintain a balance between growth, development, and adaptation to environmental challenges. Beyond their role in metabolism, isoprenoid intermediates also function as signaling molecules, linking growth regulation with stress responses [61]. In addition, plant-specific organelles such as chloroplasts further enhance this responsiveness, allowing fine-tuned adjustments of cellular metabolism to both internal and external cues [62]. Maintaining this balance is especially important for sustaining adequate pools of prenyl diphosphates, which are produced by two distinct pathways: the mevalonate (MVA) pathway in the endoplasmic reticulum (ER)/cytosol/peroxisomes and the methylerythritol phosphate (MEP) pathway in plastids [61,63]. The biosynthesis of prenyl groups used for protein modification is likewise subject to this regulatory coordination. Thus, in tobacco BY-2 cells, prenyl diphosphates derived from deoxy-xylulose phosphate (DXP) serve as substrates for protein prenylation [39], in a manner similar to that observed in apicomplexa [64]. Especially, the MEP pathway generates prenyl substrates used for the modification of a GFP sensor harboring a geranylgeranyl-specific CviL motif [40]. The question that arises is to assess to what extent MVA-derived GGPP may be generated and used by PPTs in plants. Insight can be gained by examining the organization of the gene family encoding plant GGPP synthases (GGPPS). In *Arabidopsis thaliana*, GGPPS-like enzymes are encoded by a multigenic family comprising 12 members and producing proteins targeted to diverse cell compartments, including plastids, the ER or mitochondria [65]. Particular attention was paid to characterize corresponding enzyme activities. Interestingly, cytosolic enzymes do catalyze the formation of C_25_ sesterterpene C_25_ geranylfarnesyl diphosphate (GFPP), rather than that of C_20_ GGPP [66]. In addition, isoforms catalyzing GGPP formation are exclusively localized in plastids [66], with one notable exception: *At4g36810* (*AtIDS11*), which encodes both a plastid-targeted and a cytosolic protein, expressed specifically during embryo development and likely involved in ubiquinone biosynthesis [67]. One would therefore tend to conclude that the metabolic origin of GGPP used to modify protein in plants is specifically of plastidial origin. In such a scenario, only farnesylated proteins would use MVA-derived precursors: the C_15_-FPP. This was recently confirmed in *Catharanthus roseus*, where it has been proposed that a plastid-localized CrGGPPS2 is critical for the biosynthesis of GGPP needed to modify proteins required to induce the biosynthesis of monoterpene indole alkaloids (MIAs) [44]. Two geranylgeranylated ROP GTPases (CrROP3 and CrROP5) and three regulatory proteins are modulating these MIAs biosynthesis [68,69]. This implies that the transport of prenyl diphosphate substrates from plastids to the cytosol is necessary to enable protein prenylation in the cytosol. The key unresolved question is how prenyl diphosphates cross the plastidial membrane [40,70] to serve as substrates for a putative cytosolic PGGT enzyme complex. Collectively, these observations support a plastidial origin of GGPP used for protein prenylation in plants, at least in *Arabidopsis thaliana*, *Catharanthus roseus* and tobacco cells.

### 2.2. Prioritize Substrate Availability

It can be assumed that the availability of prenyl diphosphates, the substrates of PPTs, plays a regulatory function in protein prenylation. This aspect has been mainly investigated in animal systems; therefore, we aim to broaden the perspective by comparing data across different kingdoms. Both farnesyl diphosphate (FPP) and geranylgeranyl diphosphate (GGPP) are key intermediates in the biosynthesis of numerous isoprenoids, including the abundant FPP-derived phytosterols and GGPP-derived carotenoids [63]. In a 1990 study, Manne et al. [71] evaluated PFT activity across various mammalian tissues and observed marked inter-tissue differences. Moreover, quantification studies in mammals have shown that the FPP/GGPP ratio and metabolite concentrations vary depending on tissue type [72]. It can therefore be hypothesized that abnormal biosynthesis of FPP and/or GGPP may disrupt the balance between farnesylated and geranylgeranylated proteins. Such disruptions could contribute to the development of pathological conditions, including nonalcoholic fatty liver disease [73]. Although it is unproven, a variation in this ratio may induce specific phenotypes in plants. This implies substantial substrate competition within the isoprenoid biosynthetic network by maintaining the relative ratio of prenyl diphosphates at a constant level. In contrast to other metabolic branch points, protein prenylation and thus PPTs require only small amounts of prenyl diphosphates to modify specific target substrates. This raises the intriguing question of how the cell ensures a fair allocation of limited resources, essentially, how to implement a “fair cake-cutting” strategy that satisfies diverse metabolic demands. For example, how does PFT successfully recruit FPP, despite FPP being largely consumed by squalene synthase (SQS), which catalyzes the first committed step toward phytosterol biosynthesis? On a molecular level, very little is known in plants about how such substrate competition is resolved or regulated. But recently, a sophisticated mechanism has been described in human cells that redirects the MVA pathway flux from FPP-sterol biosynthesis towards GGPP biosynthesis required for the activity of an aromatic prenyltransferase. Specifically, the aromatic prenyltransferase UBIAD1, involved in vitamin K2 biosynthesis by catalyzing the transfer of geranylgeranyl groups to 1,4-naphtoquinone menadione, is a key player in reactivating 3-hydroxy-3-methylglutaryl-CoA reductase (HMGR) activity of ubiquitinated proteins dedicated to be proteolyzed in response to high sterol contents [74]. As long as GGPP concentrations do not reach a certain threshold, the protein interacts with HMGR, ready to be proteolyzed by ER-associated degradation (ERAD). When this threshold is reached, the GGPP-protein complex is translocated to the Golgi apparatus via COPII, where it functions as a prenyltransferase for the production of vitamin K2. As a consequence, ubiquitinylated HMGR can be degraded. Whether similar regulatory mechanisms occur in plants, and moreover, in the adjustment of protein prenylation, is unknown.

Other answers may also be found in the cellular organization of metabolic pathways. Plants are able to prioritize metabolic synthesis, with, among other things, an organization into metabolons [75,76]. Such a stable soluble supramolecular organization, including all nine enzymes, has been evidenced for the biosynthesis of ubiquinone Q8 in bacteria (*Escherichia coli*), a prenylated quinone [77]. In plants, such structures appear to be predominantly membrane-associated, relatively unstable, and highly dynamic, which makes them difficult to isolate and characterize [78]. On the other hand, these dynamic complexes can be assembled on demand, i.e., according to metabolite requirements [79]. Notably, HMGR, the key enzyme in the MVA pathway, is the only ER membrane-bound eukaryotic enzyme in the pathway, while all other proteins are soluble or present in peroxisomes [80]. This unique localization suggests that HMGR may serve as a scaffold or anchoring point for the assembly of such dynamic, membrane-associated complexes. This spatial organization aligns with the concept of metabolic channeling, as proposed by Chappell [81], whereby in plants, intermediates are directly transferred from one enzyme to another within a multi-enzyme complex, minimizing diffusion into the cytosol. In the context of isoprenoid biosynthesis, metabolic channeling is thought to enhance pathway efficiency, protect labile intermediates, and allow for rapid metabolic adaptation to fluctuating cellular demands, such as during pathogen attacks. For instance, it has been proposed that, when challenged by fungal pathogens, *Solanaceae* species suppress phytosterol biosynthesis via inactivation of SQS, thereby redirecting metabolic flux toward the production of FPP-derived sesquiterpenoid phytoalexins (for review [75]). Accordingly, it has been proposed that metabolic channeling may play a key role in the redistribution of isoprenoid precursors, enabling the shift from primary metabolism (phytosterols) to defense-related specialized metabolites (phytoalexins) [81]. This allows a reorientation of the metabolic pool to sesquiterpenoid at the expense of sterol neo-biosynthesis. This type of metabolite channeling is likely closely associated with the formation of metabolons [82]. Although nearly nothing is known about PPTs’ regulation in plants, one could imagine that such a type of regulation may also occur for protein prenylation to some degree. Finally, another reason may be the impact of distinct enzymes’ properties and efficiencies of reaction rates. It is difficult to compare kinetic parameters of PPTs to those of SQS or phytoene synthase (PSY), the first branch-specific enzyme for carotenoid biosynthesis, because values have been obtained using different assays, different experimental conditions, and different manipulators. Nevertheless, according to data collected via BRENDA (https://www.brenda-enzymes.org, accessed on 16 July 2024), it appears that plant PPTs are between 10 and 50-fold more affine to prenyl diphosphates than condensation enzymes such as SQS and PSY are. This can suggest that certain prenyl diphosphates may be preferentially directed toward PPTs rather than other enzymes in the pathway. By engineering *Arabidopsis* plants to produce GGPPS-PSY fusion proteins, the metabolic flux could be redirected for carotenoid biosynthesis at the detriment of other isoprenoids [83]. Driving the expression of this minimal synthetic metabolon significantly enhances the production of the final products. More recently, it has been proposed that prenylation can be involved in metabolon formation [84].

Prioritizing the dedicated substrate is not always feasible. In cases of substrate limitation, an alternative substrate may be utilized. It has long been described that when one prenyl diphosphate substrate becomes limited, the other can serve as a substitute. A prominent example is the human oncogene protein family p21RAS, which is farnesylated. In the presence of specific PFT inhibitors, RAS proteins have been found to be modified by geranylgeranyl, allowing RAS to stay biologically active and render inhibitors inefficient [85]. Other resistance mechanisms have been described in *Plasmodium falciparum* involving amino acid substitution in the β-subunit of farnesyltransferase [55]. In tobacco BY-2 cells, an intriguing observation was reported by Randall et al. [86]. Incorporation studies using [^3^H]MVA in cells treated simultaneously with increasing concentrations of lovastatin (mevinolin), an inhibitor used to block endogenous MVA production, showed that incorporation rates decreased above 1 µM lovastatin. This suggests that an MVA-derived metabolite may positively influence PPT activities in plants, and/or that exogenous MVA is preferentially channeled toward biosynthetic pathways other than protein prenylation. Another noteworthy finding in tobacco BY-2 cells concerns the modification of MEP pathway-derived geranylgeranylated proteins [43]. Given the existence of prenol salvage pathways [36,87], prenols such as Fol and GGol can serve as precursors. After undergoing a double phosphorylation process to form FPP and GGPP, respectively, these molecules can be incorporated into proteins [60]. Protein prenylation following fosmidomycin-induced inhibition of the MEP pathway, only GGol, but not Fol, can rescue prenylation in the presence of lovastatin (mevinolin) [43]. It has been proposed that Fol (or a metabolite thereof) is not incorporated as FPP, but instead acts as a central regulator of isoprenoid biosynthesis in plants. Specifically, Fol appears to stimulate the use of an MVA-derived prenyl group for the modification of a protein that, under normal conditions, is prenylated with an MEP-derived prenyl group [43]. Altogether, these observations highlight a remarkable metabolic flexibility that enables the utilization of prenyl groups typically unused under standard conditions, alongside complementary engagement of both the MVA and MEP pathways.

### 2.3. Cross-Talk Between the MVA and MEP Pathways Mediated by Protein Prenylation

It is evident that plants possess a unique regulatory framework for protein prenylation, shaped by the dual biosynthetic origins of isoprenoid precursors via the MVA and MEP pathways. This duality grants flexibility not only in the control of prenyl group production but also in the modulation of their availability as substrates for prenyltransferases. Such metabolic adaptability may confer significant advantages under varying physiological and environmental conditions. A prominent example is during plant defense responses, where a wide range of protective mechanisms is activated, including the upregulation of specialized (secondary) isoprenoid metabolism [88]. In these scenarios, the diversion of isoprenoid precursors toward secondary metabolite synthesis may compete with protein prenylation, dynamically influencing cellular responses to stress.

**Figure 3:**
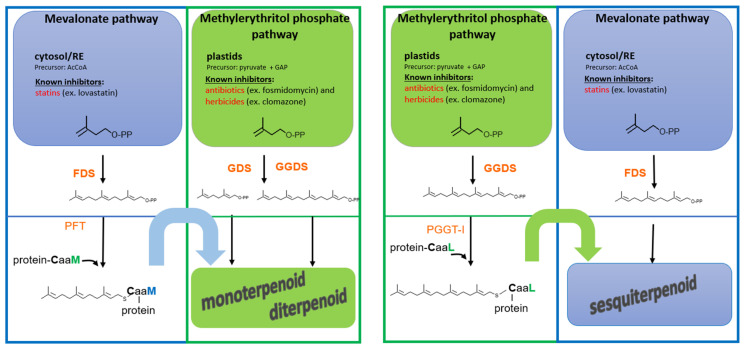
Model of functional cross-talk between the mevalonate (MVA, in blue) and methylerythritol phosphate (MEP, in green) pathways mediated by protein prenylation involved in signaling. The MVA pathway (cytosolic) and the MEP pathway (plastidial) both contribute to the biosynthesis of isoprenoid diphosphates (FPP, GGPP), which serve as substrates for protein prenylation, but also for isoprenoid biosynthesis. Prenylated signaling proteins can, in turn, activate downstream transcriptional or metabolic programs that result in the biosynthesis of specialized metabolites. Interestingly, this regulatory link allows for scenarios where an MVA-derived metabolite depends on a MEP-derived prenylated signaling protein, or vice versa, thereby introducing a novel layer of functional cross-talk between the two pathways. Pharmacological inhibition of either pathway (e.g., by statins or fosmidomycin) can disrupt this interplay, potentially blocking the synthesis of metabolites that do not directly derive from the inhibited pathway.

The metabolic origin of many of isoprenoid defense-related compounds has been extensively examined, yet it remains complex and, at times, paradoxical. For instance, taxol biosynthesis in Taxus species is considered MVA-independent [89], yet treatments with statins inhibiting HMGR in the MVA pathway lead to a downregulation of the diterpene biosynthesis [90]. A similar pattern has been observed in the biosynthesis of MIAs in *Catharanthus roseus*, which originate from the MEP pathway [91], yet are likewise inhibited by statins [92]. Conversely, the synthesis of MVA-derived sesquiterpenoids, such as capsidiol, is unexpectedly blocked by the MEP-pathway-specific inhibitor fosmidomycin [42]. While these findings may initially appear contradictory, they can be reconciled when considering the broader signaling context that governs metabolite production. In particular, these effects may be mediated through signaling components, such as small GTPases or other regulatory proteins that require prenylation for activity (Figure 3). In this model, the production of an MVA-derived metabolite may depend on a MEP-derived prenyl group used to modify a protein that plays a role in activating the corresponding signaling pathway. Conversely, the biosynthesis of a MEP-derived compound such as MIAs may rely not only on a geranylgeranylated protein (see Section 2.1. A double metabolic origin in plants), but also on an MVA-derived prenylated protein to initiate or sustain the necessary transcriptional response. This suggests a previously overlooked mode of cross-talk between the MVA and MEP pathways: not merely through the metabolic exchange of isoprenoid precursors, as is commonly understood, but through functional interdependence at the level of protein prenylation and signal transduction. More recently, we proposed that MVA acts as a sensor, triggering a metabolic cross-talk by mandating the use of an MVA-derived prenyl diphosphate, instead of a MEP-derived one, to modify a CaaL-motif protein [45]. Such a mechanism introduces a further layer of regulatory complexity and could explain the paradoxical outcomes seen when either pathway is inhibited during defense-related metabolite production. Of course, the key question is what is the benefit of such a combination? Compartmentalized metabolic regulation may provide several benefits in the context of plant defense. By utilizing distinct biosynthetic origins—the MVA and MEP pathways—for signaling components and end-product metabolites, plants can minimize metabolic competition for precursors. This spatial and functional separation enables rapid and efficient production of defense-related metabolites, particularly under stress conditions where metabolic resources must be redirected swiftly. Moreover, this arrangement can amplify the defense response. For instance, when the synthesis of a specialized isoprenoid metabolite is triggered in response to elicitation, and its activation requires a geranylgeranylated protein derived from the alternate pathway, the induction process does not deplete the same pool of precursors used for metabolite biosynthesis (Figure 3). This division of metabolic labor ensures that signaling and biosynthesis, both requiring isoprenoid precursors, can proceed in parallel without bottlenecks and/or competition, enabling a rapid and potentially exponential accumulation of protective compounds during pathogen attack or environmental stress.

### 2.4. Substrates of Prenyl Diphosphate Beyond FPP and GGPP

A versatile and adaptable mechanism of protein prenylation suggests that PPTs may recognize and harness substrates other than the canonical FPP or GGPP. One hypothesis to consider is that prenylated proteins belong to the broader class of lipid-modified proteins. In eukaryotes, many proteins are modified by both an acyl and a prenyl group, each conferring specific functional properties [93,94]. Acylation includes modifications such as myristoylation and palmitoylation. It cannot be excluded, as suggested by Sorek et al. [95], that acyl modifications may be sufficient to support the survival of KO mutants in plants, even though the *Arabidopsis* *plp* KO mutant exhibits severe developmental defects and poor viability.

Alternatively, plants synthesize a broad variety of prenyl diphosphates structurally similar to FPP and GGPP, which may serve as alternative substrates for PPTs. Prenyl group chain length varies from one isoprene group (C_5_) to several thousand. The biosynthesis catalyzed by specific prenyl or isoprenyl diphosphate synthases was largely reviewed in recent years [96,97,98,99]. It can be expected that they could be used as substrates as long as they are activated with a pyrophosphate group. As prenyl substrate donors, plants are particularly fascinating because they synthesize a fairly broad range of these molecules [100]. This hypothesis implies that the enzyme’s acceptor pocket must be suitably dimensioned to efficiently accommodate such prenyl groups. This ability may arise from conformational shifts in the protein, possibly triggered by PTMs. In addition, these alternative prenyl groups are likely modulating the affinity to the membrane, depending on the size and branching of the chain. Finally, it cannot be ruled out that alternative prenyl diphosphate substrates may act as natural competitive inhibitors of PPTs, as observed with certain synthetic analogs specifically designed for this purpose [37,101].

**Figure 4:**
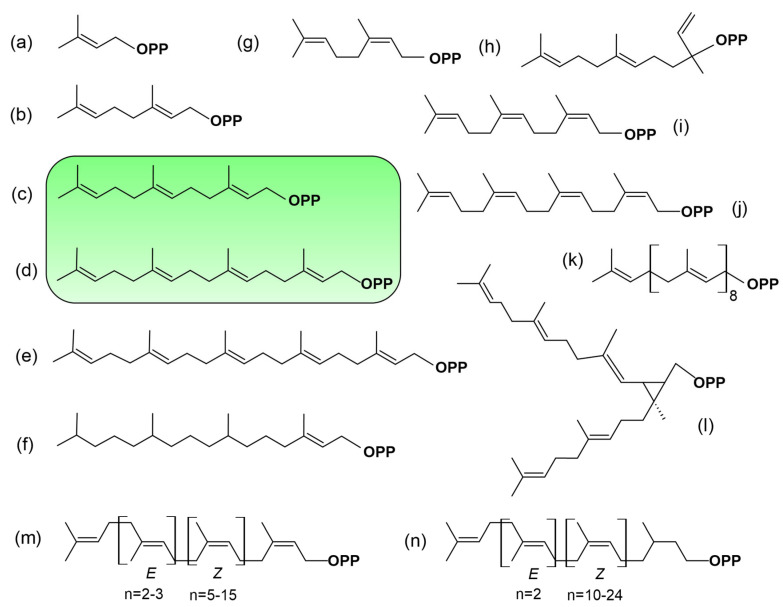
Chemical structures of some prenyl diphosphates found in plants. (**a**) C5-dimethylallyl diphosphate, (**b**) C10-geranyl diphosphate, (**c**) C15-2E,6E-farnesyl diphosphate, (**d**) C20-geranylgeranyl diphosphate, (**e**) C25-geranylfarnesyl diphosphate, (**f**) C20-phytyl diphosphate, (**g**) C10-neryl diphosphate, (**h**) C15-nerolidyl diphosphate, (**i**) C15-2Z,6Z-farnesyl diphosphate, (**j**) nerylneryl diphosphate (**k**) C45-solanesyl diphosphate, (**l**) C30-presqualene diphosphate, (**m**) polyprenols, (**n**) dolichols. In green are highlighted the structures of FPP and GGPP, the metabolites used for the basic modification of proteins.

Figure 4 presents selected examples that we consider relevant to a thoughtful discussion, illustrating the diversity arising from the modular nature of C_5_-isoprene building blocks. FPP (Figure 4c) and GGPP (Figure 4d), the canonical prenyl diphosphate substrates, are *all-trans*-prenyl diphosphate diastereomers. Shorter allylic substrates (C_5_, C_10_) are accepted by aromatic prenyltransferases [102], but there is no convincing evidence that DMAPP (Figure 4a) or GPP (Figure 4b) serve as PPT substrates. Short-chain cis-prenyl diphosphates function mainly as precursors for specific, specialized (secondary) metabolites, and their occurrence is restricted to certain plant species [100]. For instance, in wild tomato, an IDS promotes the production of (*Z*,*Z*)-FPP (Figure 4i), neryl diphosphate (NPP) (Figure 4g), nerylneryl diphosphate (NNPP) (Figure 4j) (for review [103]). These membrane-bound enzymes are located in plastids and use MEP pathway-derived substrates [104]. In a broader scale, it cannot not be excluded that under specific physiological conditions, when *all-trans*-FPP or GGPP are limited, this prenyl diphosphate might serve as a substrate. However, no experimental evidence currently supports this possibility. More interestingly, the postulate that C_25_ prenyl diphosphates (Figure 4e), precursors of sesterterpenoids [98], could also serve as substrates highlights a potentially overlooked mechanism. This idea warrants attention for several reasons. As mentioned earlier, the biosynthesis of these molecules is catalyzed by GGPPS-like enzymes located in the cytosol [66], which is also the presumed localization of PPTs. Furthermore, geranylgeranyl-derived linear molecules are produced by certain plant species, particularly in brown algae such as *Bifurcaria bifurcata* [105]. Once phosphorylated, such compounds could potentially serve as alternative substrates for PPTs or, as mentioned earlier, act as competitive inhibitors, interfering with the normal prenylation process. The fact is that this hypothesis is not only a matter of speculation; there is increasing evidence supporting the proposition that alternative substrates can indeed be used for protein prenylation.

**Figure 5:**
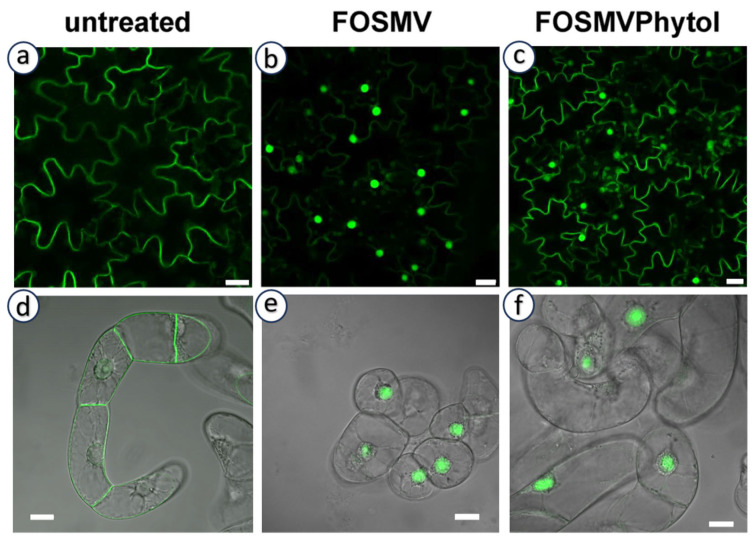
Chemical complementation of protein prenylation inhibition in green tobacco tissues. Confocal microscopy images of tobacco leaf epidermal cells (upper (**a**–**c**) panels) and non-photosynthetic BY-2 cells (lower (**d**–**f**) panels) expressing the GFP-CaM61-CVIL reporter protein. Under standard conditions, the reporter localizes to membranes (**a**,**d**) due to successful geranylgeranylation. Inhibition or absence of prenylation leads to nucleolar accumulation of the GFP signal (**b**,**e**). Upon exogenous phytol feeding, membrane localization is partially restored in chloroplast-containing leaf cells (**c**), suggesting phytol or its derivatives can serve as alternative prenylation substrates. No such rescue is observed in BY-2 cells (**f**), indicating that phytol-based complementation is dependent on photosynthetic cell metabolism. White scale bars = 20 µm.

In addition to the canonical C_15_ farnesyl and C_20_ geranylgeranyl groups, analyses of prenylated proteins have revealed the presence of non-classical isoprenoid modifications. Importantly, they were not inferred from enzyme substrate promiscuity, but rather from the direct detection of proteins carrying isoprenoid moieties of alternative chain length or structure. This demonstrates that PPTs can generate products modified by alternative isoprenoid moieties in vivo, thereby broadening the biochemical diversity of protein prenylation. In particular, phytyl (derived from the structure described in Figure 4f) [106,107], as well as dolichols (derived from structures described in Figure 4n) and polyprenols (derived from structures described in Figure 4m) have been reported [108,109]. Both dolichols and polyprenols are at least partially generated in plastids through the MEP pathway [110]. It should also be mentioned that phytol, a lipid anchor for chlorophyll in chloroplast membranes, is recycled and used as a substrate for tocopherol (vitamin E) biosynthesis via the action of a homogentisate phytyltransferase (locus *At2g18950*) [111]. However, the identity of proteins modified by these isoprenoids remains unknown, and no clear link to canonical CaaX-motif proteins has been established. This is mainly due to technical limitations: current analytical methods used to identify prenyl groups often do not preserve the associated proteins. This raises the hypothesis that either these proteins are constitutively prenylated by non-canonical isoprenoids, or that PPTs become less selective under prenyl diphosphate scarcity, enabling the use of alternative substrates. As proof of concept for this hypothesis, we tested the possibility in tobacco expressing the geranylgeranylatable GFP-Cam61-CVIL reporter protein [40], which is membrane-localized under standard culture conditions (Figure 5a,d). In the absence of prenylation, the reporter mislocalizes to the nucleolus (Figure 5b,e), reflecting a failure of membrane anchoring. Interestingly, this phenotype was partially rescued by exogenous phytol feeding [112], indicating that phytol or a phytol-derived metabolite can, at least in part, substitute for conventional prenyl donors in promoting protein prenylation (Figure 5c). However, this complementation effect was not observed in non-photosynthetic tobacco BY-2 cells (Figure 5f), suggesting that phytol metabolism and its conversion into prenylation-competent derivatives likely depend on plastidial or photosynthetic pathways, which is consistence with the study published by Parmryd et al. [107].

These findings suggest that the incorporation of phytol as a functional prenylation substrate is restricted to chloroplast-containing (chlorophyllous) cells and remains incomplete, implying that phytol-derived prenyl diphosphates might have limited affinity for PPTs or are produced at suboptimal levels. This supports the idea that plastidial metabolism contributes to the diversity of isoprenoid-derived substrates available for protein prenylation and highlights a context-dependent flexibility of the prenylation machinery.

In metazoans, two striking examples of protein modifications involving isoprenoid molecules have been described, but they have not been confirmed to occur in plants. The first involves the Hedgehog (Hh) signaling protein family, which plays an essential role in embryonic development. Hh proteins undergo an unusual self-catalyzed endoproteolysis coupled to C-terminal sterylation, whereby cholesterol is covalently attached to the processed Hh fragment [113,114]. This modification, together with N-terminal palmitoylation, is critical for the proper secretion, distribution, and signaling activity of Hedgehog proteins. The reaction begins with a rearrangement of the amide bond between a glycine and a cysteine via an N→S acyl shift. Subsequently, the 3β-hydroxyl group of cholesterol attacks the thioester intermediate to form a cholesterol–Hh adduct, which then cleaves to release a cholesterol-esterified Hh-N morphogen and Hh-C fragment [3,115]. The second example is the modification by isopentenyl adenosine (IPA), a molecule also known in plants as a cytokinin phytohormone [116]. IPA counteracts the inhibition of cell division caused by the HMGR inhibitor mevinolin/lovastatin [117,118], likely through its ability to induce the expression of cell cycle–related genes. However, these studies did not address whether IPA-modified proteins play a role in this response. We attempted to characterize such IPA-modified proteins and the associated enzyme activities in plant extracts, but these assays failed (unpublished data).

Taken together, these observations suggest that the identity and usage of alternative isoprenoid substrates may be organism-specific, potentially depending on the capacity of the organism to accumulate certain isoprenoid molecules not required for essential pathways.

## 3. CaaX Protein Substrates

Substrate flexibility in protein prenylation is not limited to the type of prenyl diphosphate donor, but also extends to the protein substrate itself. In the case of type I protein substrates, it was long assumed that specific CaaX motifs dictate the prenylation outcome—guiding the enzyme to catalyze either the transfer of a farnesyl group (farnesylation), mediated by PFT, or a geranylgeranyl group (geranylgeranylation), mediated by PGGT-I [2]. At first Ca_1_a_2_X motifs were defined as “C” corresponding to a prenyl-modified cysteine, “a1 and a2” to aliphatic amino acids (P, A, L, V or I) and X orientating the nature of the prenyl group used to modify the protein. This rule, known as the CaaX paradigm, claims that farnesylation is favored when the last amino acid X is a methionine, a serine, a cysteine, an alanine or a glutamine, while geranylgeranylation is prioritized when X is a leucine, isoleucine or a phenylalanine [52,119]. The list of amino acids compatible with prenylation was later expanded: for PFT, to include cysteine, asparagine, phenylalanine, and threonine; and for PGGT-I, to methionine and isoleucine, as well as—though to a lesser extent—valine, cysteine, and tyrosine [53]. However, the story is not as simple. Significant efforts have been made to refine our understanding of CaaX protein substrates, and the canonical Ca_1_a_2_X motif has since been reevaluated in various organisms. It is now widely accepted that this motif alone is too restrictive and does not fully reflect the diversity of recognized substrates. Although hundreds of protein substrates must be recognized by PFT or PGGT-I, substrate specificity appears to rely on structural and sequence features that go beyond the classical CaaX signature. In particular, animal PPTs recognize additional upstream residues that can influence prenylation efficiency. These upstream amino acids may promote dual prenylation by reducing the efficiency of farnesylation to a level similar to that of geranylgeranylation. Consequently, peptide specificity is driven more by catalytic reactivity than by simple binding affinity [53]. Thus, based on enzyme assays in vitro, it is now largely accepted that the CaaX paradigm of protein substrate recognition does not sufficiently describe the molecular recognition by PPTs, particularly that of mammalian PFT [51]. Indeed, PFT appears as a more flexible enzyme than PGGT-I [120,121,122]. To define the prenylome, regrouping all modified proteins, researchers applied chemical proteomics [123]. Equivalent studies of plant prenylome have not been undertaken, but the technology has been successfully applied to identify modified proteins in *Arabidopsis* cell cultures [124,125]. The classification of substrate proteins was more recently enlarged, and is out of the strict CaaX dilemma [121,126]. In fungi and mammals, aliphatic properties of a_1_ and a_2_ are not exclusive and C(x)_3_X were found to serve as substrates [120,121,126,127,128]. However, in plants, these issues are not well characterized and are actually poorly investigated.

### 3.1. CaaX Motif Proteins in Arabidopsis thaliana

Since the analytical characterization of protein prenylation is quite challenging, only a limited number of CaaX proteins have been confirmed to undergo prenylation. For plants, evidence of this modification has primarily come from radiolabeled MVA incorporation studies [129], in vitro assays [68], and chemical bioorthogonal screenings [125]. Bioinformatics offers a powerful complementary strategy to predict potential prenylation substrates across the proteome. By leveraging conserved sequence motifs, computational approaches can help prioritize candidate CaaX proteins for further experimental validation. There are 221,210 UniProtKB entries associated with prenylation, as indicated by the keyword KW–0636 (https://www.uniprot.org/keywords/KW–0636), with 1207 of these entries reviewed by Swiss-Prot (last accessed on 20 June 2025). Of these, 909 contain a C-terminal CXXX motif, where X represents any amino acid, and 153 of these are of plant origin. *Arabidopsis thaliana*, a standard plant model, is represented by 107 entries. To obtain a more detailed view of the *Arabidopsis* data, we performed a detailed analysis leveraging publicly available resources. The *Arabidopsis* Information Resource (TAIR) [130] updates and offers downloadable protein lists for further investigation (https://www.arabidopsis.org/download/index-auto.jsp?dir=/download_files/Proteins, last accessed on 20 June 2025). For our analysis, we collected 48,265 protein sequences derived from reconstituted transcripts with AUG start codons, based on an initial download in April 2017, followed by a second update in October 2019. From this dataset, we identified 1342 proteins that feature a cysteine residue at the fourth position from the C-terminus. Notably, a considerable proportion of these proteins appear to be translation products of the same gene, indicating possible transcriptional or post-transcriptional regulation events such as alternative splicing (AS). After filtering for redundancy, 885 proteins are unique sequences. Interestingly proteins comprising the CXXX motif are products of alternative messenger variants coded by 173 distinct genomic loci. This number can be increased to 2158 when C(X)_3_X motif proteins are considered. This highlights the role of transcript diversity in expanding the pool of potential prenylation substrates and suggests a complex regulatory landscape that modulates prenylation through gene architecture and RNA processing. Validation of the candidate identified prenylation substrates will require targeted in vitro assays using recombinant prenyltransferases, as well as in vivo experiments to confirm and identify modifications under physiological conditions. Such analyses are essential to determine which of these proteins are truly prenylated, and under what cellular contexts. Furthermore, it is important to consider that some proteins predicted to be prenylated may also undergo other lipid modifications, such as *S*-acylation, on distinct cysteine residues. In fact, it has been estimated that up to 25% of proteins in *Arabidopsis* may be subject to acylation [93], suggesting potential crosstalk or competition between lipid modifications that could influence protein localization and function.

CXXX-motif-containing proteins were listed and classified into functional categories. CXXX motif-containing proteins in *Arabidopsis* exhibit a wide functional distribution across diverse biological processes, indicating the broad physiological relevance of protein prenylation. Among those, we can cite membrane-associated and trafficking functions; protein regulation and quality control; gene expression and RNA metabolism; signal transduction and regulatory networks; metabolism and cellular maintenance; environmental and nutrient responses. Vesicle trafficking is one of the most prominently represented categories, including machinery for clathrin-dependent/independent transport, COPI/COPII coatomer systems, SNARE complex components, and membrane tethering/fusion regulation. Protein translocation across organelles (e.g., chloroplasts, ER, mitochondria, peroxisomes) is also notable. These categories underscore a major role for prenylated proteins in intracellular membrane dynamics, likely through their membrane-targeting properties. Notably, a significant number of proteins are involved in protein modification (e.g., phosphorylation, lipidation, glycosylation) and protein homeostasis, including protein folding, autophagy, and the ubiquitin-proteasome system. These categories suggest that CXXX proteins are important in modulating protein function, turnover, and localization. Numerous proteins are associated with RNA biosynthesis (Pol I/II/III transcription), RNA processing (splicing, modification, surveillance), and protein biosynthesis (translation initiation/elongation/termination). This highlights the involvement of CXXX proteins in regulating gene expression at transcriptional and post-transcriptional levels. Functional bins related to phytohormone action (e.g., auxin, ABA…), G-protein signaling, TOR, and SnRK1 kinase pathways indicate that CXXX motif proteins may also play regulatory roles in developmental and environmental signaling. The presence in circadian rhythm and PCD (programmed cell death) systems further supports a signaling/regulatory role. While less dominant, CXXX proteins also participate in primary metabolism (carbohydrates, lipids, amino acids), redox homeostasis, coenzyme biosynthesis, and polyamine metabolism. This distribution implies accessory or regulatory roles in central metabolic processes. Finally, CXXX motif proteins are represented in bins for responses to light, drought, pathogens, and nutrient uptake (N, P, S, Fe, Cu), supporting their involvement in stress responses and nutrient homeostasis. Overall, CXXX motif-containing proteins in *Arabidopsis* are predominantly associated with membrane trafficking, protein regulation, and RNA metabolism, reflecting their likely roles as prenylated proteins targeted to membranes where they mediate signal transduction, protein turnover, and vesicular transport. Their widespread presence across many functional domains emphasizes the central role of prenylation in coordinating plant cellular processes.

### 3.2. Functional Conservation and Diversification of CxxX-Containing Proteins

**Figure 6:**
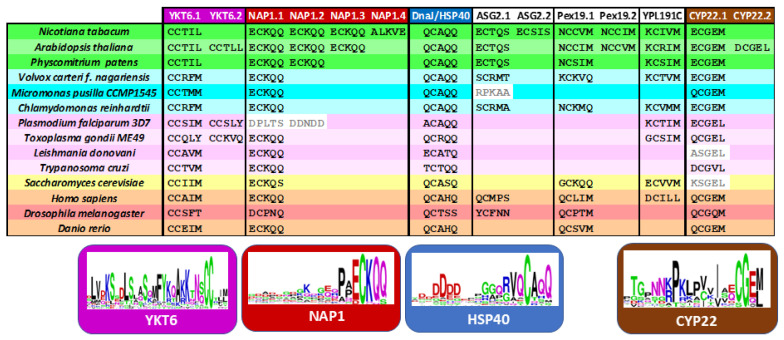
Conservation across eukaryotes of the C-terminal XCxxX motif in selected prenylated proteins. Plants are represented in green, cyanobacteria in blue, protozoa in purple, fungi in yellow and animals in orange/red colors. Sequences of YKT6, NAP1, HSP40, ASG2, Pex19, YPL191C) and CYP22 were obtained from Unipro. WebLogo analysis of the C-terminal sequences was realized on the last 30 amino acids using Version 2.8.2 (2005-09-08) of WebLogo 3 (https://weblogo.berkeley.edu/logo.cgi, accessed on 25 June 2025). The height of each letter represents the degree of amino acid conservation at that position, with larger letters indicating higher conservation. This conservation suggests strong evolutionary pressure to preserve the prenylation signal across diverse proteins.

Despite the evolutionary conservation of the prenylation process, a surprisingly large proportion of proteins containing putative CaaX-like motifs appear to be plant-specific. This suggests that, while the enzymatic machinery for prenylation is broadly conserved across eukaryotes, its substrate repertoire has diversified significantly in different organisms. Figure 6 compiles proteins that have earlier been characterized as real acceptors of prenyl groups at least in one of those organisms [33,129,131,132].

These core proteins are consistently carrying a C-terminal CXXX motif across all eukaryotic lineages: (i) YKT6 (a soluble NSF attachment protein receptor/SNARE protein), (ii) NAP1 (nucleosome assembly protein-1), (iii) HSP40 (a heat shock protein member) and (iv) CYP22 (a peptidyl-prolyl isomerase chaperone member). A WebLogo analysis of the C-terminal sequences revealed a highly conserved CaaX motif, suggesting a strong evolutionary pressure to maintain prenylation at this site (Figure 6). These shared substrates likely represent the ancestral, conserved functions of prenylation, whereas the plant-specific targets reflect lineage-specific adaptations of this post-translational modification. Interestingly, although these conserved proteins differ in function, they all contribute to protein homeostasis and regulation and are involved in cellular stress adaptation. This functional convergence highlights the essential role of prenylation in maintaining proteostasis and facilitating adaptive responses across eukaryotes.

YKT6 are SNARE proteins involved in membrane trafficking pathways such as intra-Golgi transport and autophagic membrane fusion [133]. In *Arabidopsis*, YKT61 is required for gametophyte development. Interestingly, plant proteins contain a CCxxL motif with a C-terminal leucine, while for other eukaryotes, the protein carries mainly a C-terminal methionine (Figure 6). As mentioned earlier, YKT6 is dually prenylated on its tandem cysteine motif in yeast and mammalian [31,133,134]. *Arabidopsis* contains an ortholog gene to *PTAR1*, localized at locus *At1g10095*, which could be a good α-subunit candidate to form an active heterodimeric enzyme with the RAB PGGT β-subunit [29]. HSP40 belongs to the DnaJ family of chaperones that are associated with HSP70. It is believed that each HSP70–HSP40 pair facilitates a distinct cellular process at a specific location within the cell, thereby fulfilling a defined physiological role [11]. For instance, the HSP70-4–HSP40/J3 pair plays a critical role in protecting plants against prolonged heat stress [11]. Interestingly, the farnesylated AtJ3 can interact with *E. coli* HSP70 and enhance thermotolerance in this bacterium [135], indicating that the lipid modification is crucial for its functional interaction and chaperone activity. The WebLogo analysis revealed a conserved aspartic acid-rich region in the C-terminal tail (Figure 6). Two other chaperone proteins contain CxxX motif proteins. First, the peptidyl-prolyl isomerase belongs to the cyclophilin CYP22 family [136], but proteins of the yeast *Saccharomyces cerevisiae* and the protozoa *Leishmania donovani* miss the cysteine at position −4. The third protein, NAP1, is a histone chaperone that mediates somatic homologous recombination and facilitates nucleosome assembly during stress [137]. AtNAP1 seems to affect plant growth under nitrogen conditions [138] and its farnesylation is necessary to activate cell proliferation during leaf development [130], while *Plasmodium falciparum* proteins lack a potential prenylation motif.

In conclusion, farnesylation appears to be the more ancient and evolutionarily conserved form of protein prenylation. From yeast to animals, PFTs are highly conserved in both sequence and structure across diverse eukaryotic lineages, underscoring their early evolutionary origin. Although PGGTs are also conserved but their functional divergence across taxa suggests a later emergence, likely associated with more specialized or lineage-specific roles [4,139]. Our analysis indicates that most conserved proteins possess farnesylatable CxxM/Q motifs, with the notable exception of CYP22, which features a dual-targeting motif (Figure 6). In contrast, proteins predicted to undergo geranylgeranylation are more often organism-specific, suggesting that this type of modification arose later in evolution. This pattern aligns with the central role of MVA-derived FPP in primary metabolism, where it contributes to the synthesis of essential compounds such as sterols, ubiquinone, and dolichols. Protein farnesylation is consistent with this framework, reflecting both its early evolutionary origin and its fundamental role in cellular function across diverse eukaryotic lineages.

### 3.3. Validation of CxxX-Containing Proteins as Substrates of PPT

**Figure 7:**
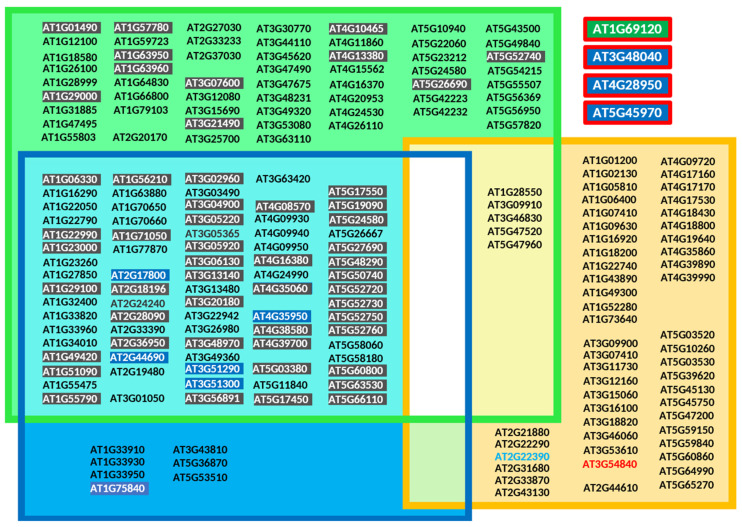
Proteins predicted to be prenylated in *Arabidopsis thaliana* according to the prenylation prediction suite PrePS [140]. To simplify access, loci position on the *Arabidopsis thaliana* genome have been indicated for each gene encoding a recognized protein substrate for PPTs and not protein accession numbers. Type I and type II (RAB) protein prenylation substrates were selected. The RAB protein (RABA4E) encoded by the gene located at locus *At2g22390* (light blue) corresponds to a pseudogene, and that of locus *At3g54840* does not contain a prenylation motif (red). It should be noted here that the current prediction PrePS site (https://mendel.imp.ac.at/PrePS/, accessed on 10 November 2024) no longer supports the identification of plant RAB-PGGT-II substrates, and that to counter this problem, the sequences can alternatively be submitted to GPS-lipid site (http://lipid.biocuckoo.org/webserver.php, accessed on 10 November 2024). All collected proteins, depending on the nature of the prediction, have been grouped into farnesylated proteins (green group), geranylgeranylated proteins (blue group) or RAB-related proteins (orange group). Proteins being predicted as farnesylated or geranylgeranylated are indicated in cyan. Loci coding for plant-specific metal-binding CaaX family proteins are highlighted in black (52 members), including the 45 proteins constituting the HIPP superfamily. Blue-highlighted loci referring to the ROP family (9 out of 11 members are prenylated). Scared in red are proteins being prenylated, but that have not been identified by PrePS.

Over the years, several prediction tools have been developed to identify potential protein prenylation substrates, evaluating the likelihood that a given protein is targeted by PFT, PGGT-I, or PGGT-II [140,141,142]. More recently, machine learning approaches have been successfully applied to address the same question [143]. Here, plant protein sequences containing CxxX motifs were analyzed using the prenylation prediction suite PrePS [140] (https://mendel.imp.ac.at/PrePS/, accessed on 10 November 2024) and classified into three groups, each corresponding to one of the PPT enzymes for which they are predicted to serve as substrates (Figure 7).

It can be concluded that those models have their limitations. Chiefly, some contradictions were identified for which experimental studies proved modification of specific plant proteins. For instance, the *Arabidopsis thaliana* gene at the locus *At1g69120* (Figure 7) encodes APETALA1, a MADS-box family transcription factor (accession number P35631), important for floral development and including a prenylatable CFAA motif essential to modulate its function [19]. PrePS does not recognize this protein and its CaaX motif as a PPT substrate. Similarly, loci *At3g48040* coding for ROP10/RAC8, *At4g28950* coding for ROP9/RAC7 and *At5g45970* coding for ROP7/RAC2 have been shown to be geranylgeranylated [95,144], but were not identified by PrePS. Among 57 loci [145] (Figure 7) encoding RAB-PGGT-II protein substrates, 5 are predicted by PrePS to be substrates of both PGGT-II and PFT (Figure 7). Nonetheless, the most reliable approach to validate a CaaX motif remains the structural characterization of the chemical group covalently attached to the cysteine residue. However, commonly used techniques are limited by their reliance on in vitro approaches; in vivo, the regulation of enzymatic activities and substrate specificities can lead to the attachment of different chemical groups. Mass spectrometry-based proteomics [146,147,148] should be adapted for use with plant material. Furthermore, prenylomes should be characterized under various physiological conditions, including different developmental stages and stress responses.

### 3.4. Post-Transcriptional Modifications as a Mechanism to Obtain New Prenylatable Proteins

**Figure 8:**
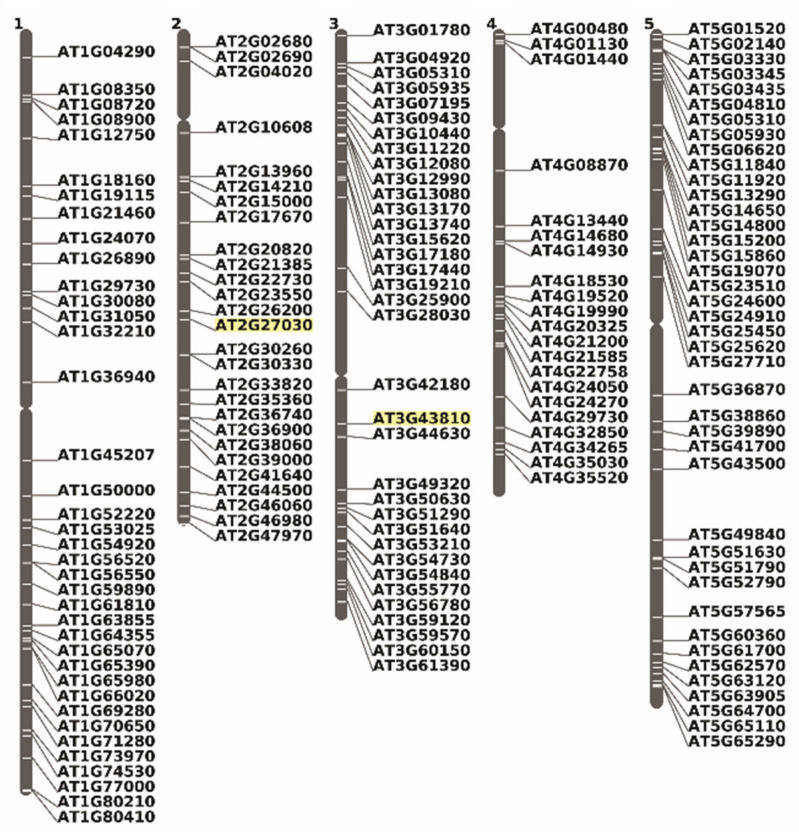
Chromosomal localization of *Arabidopsis* CaaX motif proteins, products of an alternative messenger variant. The inventory was completed manually by analyzing cDNA sequences downloaded from TAIR (https://www.arabidopsis.org/servlets/ViewChromosomes, accessed on 6 September 2022) and using the chromosome viewer tool. In yellow are highlighted the chromosomal positions of Cam5 and Cam7 on chromosomes 2 and 3, respectively.

We identified 173 genomic loci that give rise to alternative mRNA variants not found in the canonical transcriptome. These variants potentially encode proteins with CaaX-like motifs and are randomly distributed across all five *Arabidopsis* chromosomes (Figure 8).

Surprisingly, proteins encoding isoprenoid biosynthesis-related enzymes have also been identified. Among them are *At1g66020*, which encodes a terpene synthase; *At4g21200*, which encodes a gibberellin 2-oxidase; and *At5g15860*, which encodes prenylcysteine methylesterase involved in protein post-prenylation maturation. Although it has not been definitively shown that these proteins are prenylation substrates, it is reasonable to hypothesize that they undergo lipidation. In the following section, we will explore the mechanisms that have been employed to generate these mRNA variants.

#### 3.4.1. Gene Duplication

As seen earlier, specific families of CaaX protein substrates are exclusively found in plants. A prominent example is members of heavy metal-associated plant proteins (HPPs) family. These metallochaperones contain heavy metal-associated (HMA) domains containing two cysteine residues that complex the metal. Among 77 HPPs in *Arabidopsis* and 69 in rice, respectively, 45 and 59 are heavy metal-associated isoprenylated plant proteins (HIPPS) [149,150]. While their function is not yet clearly established, these metallochaperones are inducible by abiotic stresses, such as metal stress, cold, … Several co-precipitation experiments led to the isolation of some of these proteins, pointing to a role in protein complex assembly, consistent with their putative chaperone activity [151,152]. Accordingly, these molecular chaperones participate in diverse processes, ranging from heavy-metal detoxification and regulation of abiotic stress responses to plasmodesmata-mediated pathogen mobility and endoplasmic reticulum-associated degradation (ERAD) of proteins [149,153,154,155].

Within other protein families, the presence of a CaaX motif is limited to a minority of isoforms, thereby conferring functional specificity to the modified form. For instance, in the gene family coding for 6-phosphogluconolactonases (PGL), an enzyme part of the oxidative pentose-phosphate pathway (OPPP), the isogene 2 coding for PGL2 (locus *At3g49360*) encodes a protein bearing a C-terminal CSIL motif [156]. Prenylation of the protein enables colocalization with glucose-6-phosphate dehydrogenase (G6PD5.4 isoform) in ER subdomains [84]. It has to be noted here that ER-localized prenylated proteins are uncommon; they are rather localized in the plasma membrane. The alternative splice variant of G6PD5 encodes a protein with an extended N-terminus formed by two membrane-spanning domains. At this stage, it can be speculated that prenylation of PGL2 favors the interaction with G6PD5.4, and that ER-localization is controlled by this latter enzyme. Another example is HSP proteins. In *Arabidopsis* more than 100 HSP40 proteins are encoded, but only J2 and J3 are prenylated, presumptively by a farnesyl moiety [129]. It is thought that heat shock promotes prenylation of these HSP proteins, which is correlated with heat tolerance [157].

#### 3.4.2. Alternative Splicing

AS is a post-transcriptional regulatory mechanism whereby a single gene generates multiple mRNA splice isoforms through the selective use of alternative splice sites [158]. In this way, AS events contribute to transcriptome and thus to proteome diversity, especially in the context of development and immunity [159], and seem to be also a way to regulate the emergence of new CaaX prenylation motifs [160].

A notable example is provided by plant calmodulins. Calmodulins are small acidic Ca^2+^ sensor proteins. In the presence of Ca^2+^, their conformation is readjusted, allowing the association with calmodulin-binding proteins and thus controlling different cellular responses and functions [161]. In humans, a Ca^2+^-calmodulin complex interacts with the KRAS4b variant of KRAS, a farnesylated protein localized in the plasma membrane, involved in signal transductions leading to cell proliferation [162]. Through this interaction, sustained by the polybasic C-terminus region and sequestering the lipidic prenyl group, KRAS4b is translocated into the cytosol following Ca^2+^ signaling events [163]. In 1999, Rodríguez-Concepción et al. identified in petunia a calmodulin (CaM53) with an extended C-terminal basic domain and a conserved PGGT-I recognition motif [164]. It has been proposed that CaM53 can coordinate the metabolic activity of plant cells with Ca^2+^-activated pathways in the plasma membrane and the nucleus. Besides, it had been demonstrated that the extended C-terminal tail of the petunia and rice calmodulins is necessary for the protein to be recognized by PPTs. But its role extends further, as it also introduces a regulatory mechanism to fine-tune Ca^2+^ signaling events [165]. The prenylation status of CaM53 modifies the subcellular localization, with a plasma membrane localization when prenylated, while the unmodified protein localizes in the nucleus with a strong nucleolar labeling [164]. At the same time, the localization of CaM53 could also be manipulated by light and sugar, suggesting a role for CaM53 in sugar-sensing signal transduction pathways. However, experimental evidence for such a function is still not provided.

Such plant-specific CaM proteins are not restricted to petunia and rice. By carefully analyzing plant splice variants, it can be concluded that many dicotyledons and monocotyledons have adopted the same mechanisms to express prenylatable CaM proteins. *Arabidopsis* contains seven genes generating at least four different isoforms [166]. From those, two calmodulin and one calmodulin-like gene product variants coding for proteins with C-terminal extensions are described: *At2g27030.3* (Calmodulin5-Cam5), *At3g43810.2* (Calmodulin7-Cam7) and *At3g22930.2* (Calmodulin-like 11), whose sequences are accessible on Gramene resource (https://ensembl.gramene.org/Arabidopsis_thaliana/Info/Index, accessed on 6 September 2022). All exhibit the characteristic PGGT-I-specific CaaX motifs. Notably, CaM7 has been identified as a transcriptional regulator involved in light signaling pathways, promoting photomorphogenesis through its interaction with AtMYB4 and HY5 [167,168]. This finding adds to the growing list of transcription factors modified by prenyl groups, particularly in plants. A shared feature among these C-terminally extended proteins is their enrichment in positively charged amino acids (such as arginine [R] and lysine [K], and occasionally the acidic residues aspartate [D] and glutamate [E]). These residues facilitate electrostatic interactions with anionic lipids present in specific membrane structures, which are crucial for proper protein localization.

#### 3.4.3. Gene Fusion

**Figure 9:**
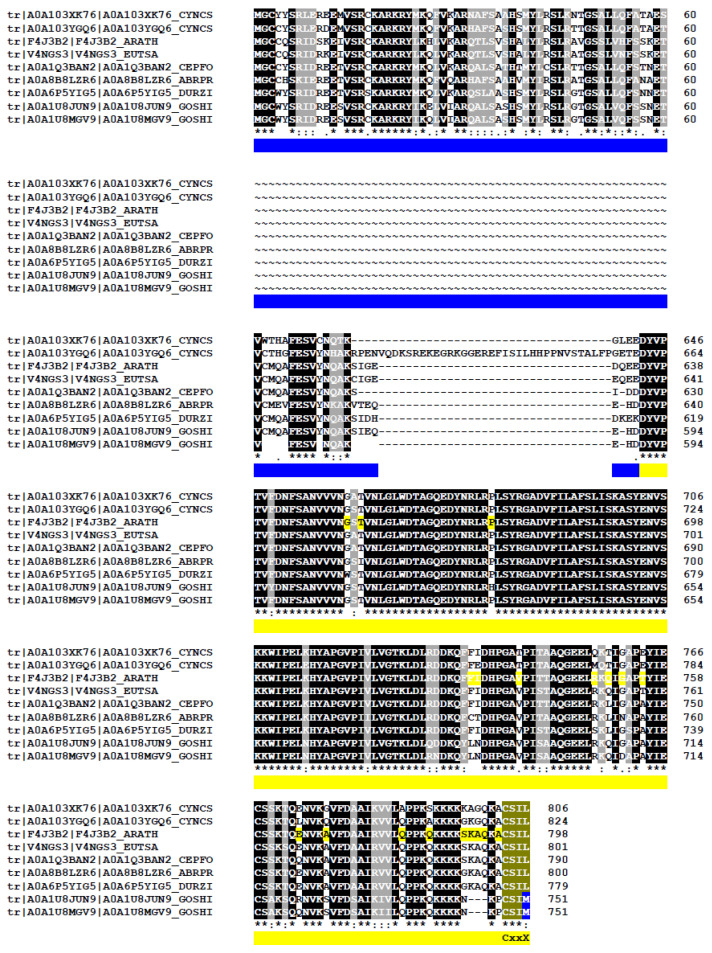
**Alignment of** APSR1-ROP1 fusion proteins found across diverse plant species. In the BLAST amino acids alignment, “*” indicates identical residues; “:” indicates conserved substitutions (strong similarity) and “.” indicates semi-conserved substitutions (weak similarity). CYNCS: *Cynara cardunculus*, *Asteraceae* (A0A103XK76; A0A103YGQ6); ARATH: *Arabidopsis thaliana*, *Brassicaceae* family (F4J3B2); EUTSA: *Eutrema salsugineum*, *Brassicaceae* (V4NGS3), CEPFO: *Cephalotus follicularis*, *Cephalotaceae* (A0A1Q3BAN2), DURSI: *Durio zibethinus*, *Malvaceae* (A0A6P5YIG5), ABRPR: *Abrus precatorius*, *Fabaceae* (A0A8B8LZR6), GOSHI: *Gossypium hirsutum*, *Malvaceae* (A0A1U8JUN9, A0A1U8MGV9). These proteins were identified through BLAST searches on the UniProt platform (https://www.uniprot.org/blast/uniprotkb/ncbiblast-R20250819-092328-0103-48514368-p1m/overview, accessed on 18 August 2025) and aligned using UniProt ClustalO. To improve readability, sequence alignments corresponding to amino acids (AA) between 61-612, using *Arabidopsis thaliana* as a reference, have been removed. Identical AA are highlighted in black, while similar residues are shaded in gray. Protein identities range from 67.53% to 97.4%. Blue and yellow lines indicate the respective delimitation of APSR1 and ROP1 in *Arabidopsis*. The C-terminal CaaX prenylation motif is indicated.

An illustrative example of a gene fusion-derived transcript variant featuring a prenylation motif is APSR1 (Altered Phosphate Starvation Response 1), a pyridoxal-phosphate-dependent serine hydroxymethyltransferase. The protein encoded by the gene at locus *At3g51290* is likely a transcription factor involved in the maintenance of the root meristem [169]. In its unfused form, APSR1 regulates the expression of the auxin transporter PIN7 under phosphate starvation conditions and plays a role in pattern specification during root development [170]. As expected for a transcription factor, the 71 kDa protein is predicted to localize to the nucleus (https://suba.live/suba-app/factsheet.html?id=AT3G51290.1, accessed on 25 April 2023). A second transcript variant (*At3g51290.2*) results from a readthrough fusion between *APSR1* and the adjacent gene *At3g51300*, which encodes Rho of Plants 1 (ROP1 or RAC11). As a standalone protein, ROP1 is a small GTP-binding protein involved in signal transduction during pollen tube tip growth [171]. The resulting 89 kDa fusion protein is predicted to localize to the cytosol. (https://suba.live/suba-app/factsheet.html?id=AT3G51290.2, accessed on 25 April 2023). However, it contains a C-terminal CSIL geranylgeranylation motif which, upon prenylation, could redirect the fusion protein to the plasma membrane—similar to what is observed in prenylated calmodulins [164]. Comparable variants were not confined to *Arabidopsis* but were also detected in eight additional mRNAs expressed in diverse species such as *Eutrema salsugineum*, *Cephalotus follicularis*, *Durio zibethinus*, *Abrus precatorius*, *Gossypium hirsutum*, *Cynara cardunculus* (Figure 9).

This list is expected to grow with the analysis of long cDNAs from diverse plant species. Interestingly, both cotton (*Gossypium hirsutum*) proteins contain a CSIM prenylation motif predicted to direct farnesylation rather than geranylgeranylation. Interestingly, both proteins in cotton (*Gossypium hirsutum*) contain a CSIM prenylation motif that should drive farnesylation rather than geranylgeranylation. The biological significance is unknown.

In conclusion, the presence of these chimeric proteins indicates that gene fusion can alter the subcellular localization of the first protein in the fusion. Moreover, ROP1 itself may have functional roles beyond its well-established involvement in pollen tube growth.

## 4. Conclusions, Open Questions, and Challenges

This review has explored several possibilities for expanding the substrate range of plant PPTs. While some ideas remain speculative, such hypotheses are valuable, as bold exploration often drives scientific progress beyond the accumulation of established facts. To date, limited attention to alternative substrates has constrained experimentation and shaped the interpretation of existing data. The discovery of new or previously overlooked prenylated proteins may depend largely on developing more sensitive and specific analytical methodologies—a major bottleneck in the field. Particular emphasis should be placed on untargeted identification of protein-bound prenyl groups. However, plant-specific features such as the cell wall, pigment-containing organelles, acidic vacuoles, and the diversity of isoprenoid metabolites make isolating lipid-modified peptides especially challenging. Thus, improved tools for detecting and characterizing prenylation will be essential to advance our understanding of its regulation in plants.

Serious consideration of substrate diversification also requires a rigorous evaluation of the substrate flexibility of PPT enzymes, together with an understanding of how their activity is regulated in vivo. Notably, protein modifications in plants depend on isoprenoid precursors derived from both the MVA and MEP pathways, a dual origin that is often overlooked in the literature. In this context, an intriguing, unresolved question is how prenylation enzymes, generally considered cytosolic [172], are able to utilize both cytosolic- and plastid-derived isoprenoid precursors, reflecting the dual origin in plant cells. A comprehensive understanding of PFT and PGGT function will require mapping their interactomes across diverse physiological conditions and identifying the complete spectrum of metabolite substrates involved. Such a broader view may also uncover regulatory mechanisms that connect metabolic flux with protein modification.

Expanding the substrate scope of protein prenylation also carries strong biotechnological potential. For instance, the engineered introduction of CaaX motifs can enable site-specific lipid conjugation of proteins through bioorthogonal chemistry, offering novel strategies for manipulating protein function, trafficking, or membrane association [173]. Recent advances have transformed PFT from a natural lipidation enzyme into a versatile bioconjugation platform [174]. By mutating enzyme active sites and designing non-natural prenyl analogues, researchers have expanded their substrate scope, enabling site-specific attachment of diverse chemical groups to proteins. This approach offers a modular, efficient, and bioorthogonal strategy for manipulating protein localization, function, and assembly, with potential applications in synthetic biology, therapeutic design, and biomaterials development [174]. Future efforts are directed toward further enzyme engineering to enhance substrate tolerance, extending these strategies to other prenyltransferases and in vivo systems, and integrating engineered prenylation with broader synthetic biology frameworks for dynamic and programmable protein control. Using a more flexible plant PPT could significantly improve the engineering potential of protein prenylation systems. Its intrinsic flexibility could allow the enzyme to accommodate non-natural prenyl donors or engineered CaaX motifs more efficiently, thereby expanding the range of proteins that can be selectively modified. Such properties could make plant enzymes powerful biotechnological tools for site-specific lipidation, bioorthogonal conjugation, or the design of programmable protein–membrane interactions, ultimately enhancing the precision and versatility of prenylation-based protein engineering strategies.

Altogether, moving beyond canonical models of prenylation not only challenges long-standing assumptions in the biochemistry of protein prenylation but also opens new directions for investigating protein modification, metabolic regulation, and applied synthetic biology. As methodologies continue to advance, the field is positioned to reveal a more complete and dynamic understanding of protein prenylation in plants and beyond.

## Data Availability

No new data were created or analyzed in this study. Data sharing is not applicable to this article.
